# Hot and cold weather based on the spatial synoptic classification and cause-specific mortality in Sweden: a time-stratified case-crossover study

**DOI:** 10.1007/s00484-020-01921-0

**Published:** 2020-04-23

**Authors:** Osvaldo Fonseca-Rodríguez, Scott C. Sheridan, Erling Häggström Lundevaller, Barbara Schumann

**Affiliations:** 1 Department of Epidemiology and Global Health, Umeå University, 901 87 Umeå, Sweden; 2 Centre for Demographic and Ageing Research, Umeå University, 901 87 Umeå, Sweden; 3 Department of Geography, Kent State University, Kent, OH 44242 USA

**Keywords:** Cardiovascular mortality, Respiratory mortality, Spatial synoptic classification, Sweden, Hot weather, Cold weather

## Abstract

The spatial synoptic classification (SSC) is a holistic categorical assessment of the daily weather conditions at specific locations; it is a useful tool for assessing weather effects on health. In this study, we assessed (a) the effect of hot weather types and the duration of heat events on cardiovascular and respiratory mortality in summer and (b) the effect of cold weather types and the duration of cold events on cardiovascular and respiratory mortality in winter. A time-stratified case-crossover design combined with a distributed lag nonlinear model was carried out to investigate the association of weather types with cause-specific mortality in two southern (Skåne and Stockholm) and two northern (Jämtland and Västerbotten) locations in Sweden. During summer, in the southern locations, the Moist Tropical (MT) and Dry Tropical (DT) weather types increased cardiovascular and respiratory mortality at shorter lags; both hot weather types substantially increased respiratory mortality mainly in Skåne. The impact of heat events on mortality by cardiovascular and respiratory diseases was more important in the southern than in the northern locations at lag 0. The cumulative effect of MT, DT and heat events lagged over 14 days was particularly high for respiratory mortality in all locations except in Jämtland, though these did not show a clear effect on cardiovascular mortality. During winter, the dry polar and moist polar weather types and cold events showed a negligible effect on cardiovascular and respiratory mortality. This study provides valuable information about the relationship between hot oppressive weather types with cause-specific mortality; however, the cold weather types may not capture sufficiently effects on cause-specific mortality in this sub-Arctic region.

**Electronic supplementary material:**

The online version of this article (10.1007/s00484-020-01921-0) contains supplementary material, which is available to authorized users.

## Introduction

The effect of low and high ambient temperature on all-cause and cause-specific mortality has been studied in different geographic areas across the world (Luan et al. 2018; Rey et al. 2007; Sheridan et al. 2019; Urban et al. 2019; Urban and Kysely 2018). In general, high and low temperatures are associated with higher risk of all-cause mortality and mortality by cardiovascular and respiratory diseases due to different pathophysiological mechanisms (Anderson and Bell 2009; Gasparrini et al. 2012; Rocklöv 2010). Previous studies in Sweden focused mainly on analysing the impact of temperature on all-cause mortality or cause-specific mortality (Astrom et al. 2013; Oudin Astrom et al. 2018; Rocklöv et al. 2014), with the effect of humidity and metrics such as apparent temperature considered in some studies as well (Rocklöv et al. 2011; Rocklöv and Forsberg 2010).

Furthermore, it has been demonstrated that not only ambient temperature but also other variables like relative humidity represent important risk factors for cardiovascular and respiratory diseases (Davis et al. 2016; Rocklöv and Forsberg 2010). There is an increasing interest on assessing the relationship between weather and human health due to the synergic effect produced by many environmental variables on health and comfort (Davis et al. 2003; Hondula et al. 2014). Additionally, other meteorological factors such as wind speed, solar radiation and air pressure can increase cardiovascular and respiratory morbidity and mortality risk (Carder et al. 2005; Ferrari et al. 2012).

While single meteorological parameters (solar radiation, temperature, humidity, wind speed, atmospheric pressure etc.) influence human health to different degrees, they have also been shown to exert a collective effect (Driscoll 1990; Vitkina et al. 2018; Yarnal 1993). Thus, to assess the impact of weather on human health, different metrics have been applied. Among these are humidex (Masterton and Richardson 1979), wind chill (Osczevski and Bluestein 2005), apparent temperature (Steadman 1979; Steadman 1984), Universal Thermal Climate Index (UTCI) (Jendritzky et al. 2012), excess heat factor (Nairn and Fawcett 2015) and the gridded weather typing classification (Lee 2015a, b).

The spatial synoptic classification (SSC) proposes a different and complementary framework for analysis. Because of its holistic nature, the SSC allows testing of the assumption that a synergistic, concomitant influence of multiple weather variable mechanisms may impact the physiological response to weather (Hondula et al. 2014; Sheridan 2002). This different framework highlights theoretical advantages of the SSC compared with the simple temperature model (Hondula et al. 2014).

The SSC classifies daily weather conditions into one of the following seven weather types: dry polar (DP), dry moderate (DM), dry tropical (DT), moist polar (MP), moist moderate (MM), moist tropical (MT) and transition (TR) (Sheridan 2002). The classification is based on several weather variables as follows: air temperature, dew-point temperature, sea-level pressure, wind speed and cloud opacity measured every 6 hours. The SSC weather types represent a holistic categorical assessment of the daily weather conditions at specific locations. More information about the methodology is available in Sheridan (2002).

Moreover, it must be considered that the relationship between weather and health varies in space (Gosling et al. 2007; Hajat et al. 2010) and time (seasons) (Hondula et al. 2014; Sheridan 2002). Because the SSC is a relative rather than an absolute classification system (Hondula et al. 2014; Sheridan 2002), it has therefore been used in many studies to evaluate weather–health effects on the population, including mortality and morbidity (Hondula et al. 2014).

Beyond the conditions on each day individually, another important aspect to consider in studies of weather and health is the duration of heat and cold events. A number of studies have shown the association of duration of heat and cold events and a large excess in mortality (Anderson and Bell 2009; Barnett et al. 2012; Rocklöv et al. 2014; Smith and Sheridan 2018). The prolonged exposures to hot or cold extreme weather conditions over several consecutive days could produce physiological exhaustion related to cumulative stress, increasing health problems and mortality (Rocklöv et al. 2014).

Based on the SSC, oppressive weather can be identified as weather types that significantly increase the mortality above the specific seasonal baseline (Sheridan et al. 2009). We have previously shown the association between all-cause mortality and oppressive hot (DT and MT) and cold (DP and MP) weather during summer and winter, respectively, in southern and northern locations in Sweden (Fonseca-Rodríguez et al. 2019). Differences in mortality associated with oppressive weather were found between southern and northern locations and between seasons. The dry tropical (DT) and moist tropical (MT) weather types were the most harmful oppressive weather types, showing a cumulative relative risk (RR) over 14 days of 1.08 (95%CI, 1.02–1.14) and 1.05 (1.01–1.10), respectively, and affecting mostly the southern locations during the summer. Even the moist polar (MP) weather types in summer had an impact on mortality in the southern study areas, with RR of 1.05 (1.01–1.09), but in winter, the dry polar (DP) increased the mortality risk (1.05; 1.01–1.09) over 28 days. Conversely, the northern study locations were less affected by oppressive weather types in both summer and winter. However, it is important to investigate the effect of different weather types on cause-specific mortality in order to bring more specific results to policymakers.

In this study, we expanded upon our previous work (Fonseca-Rodríguez et al. 2019) to assess the impact of different SSC weather types on specific causes of death in different regions of Sweden. In particular, we aimed at (a) assessing the effect of hot oppressive weather types and the duration of heat events (DT or MT days in sequence) on cardiovascular and respiratory mortality in summer and (b) examining the effect of cold weather types and the duration of cold events (DP and MP days in sequence) on cardiovascular and respiratory mortality in winter.

## Materials and methods

### Study locations and data

**Fig. 1:**
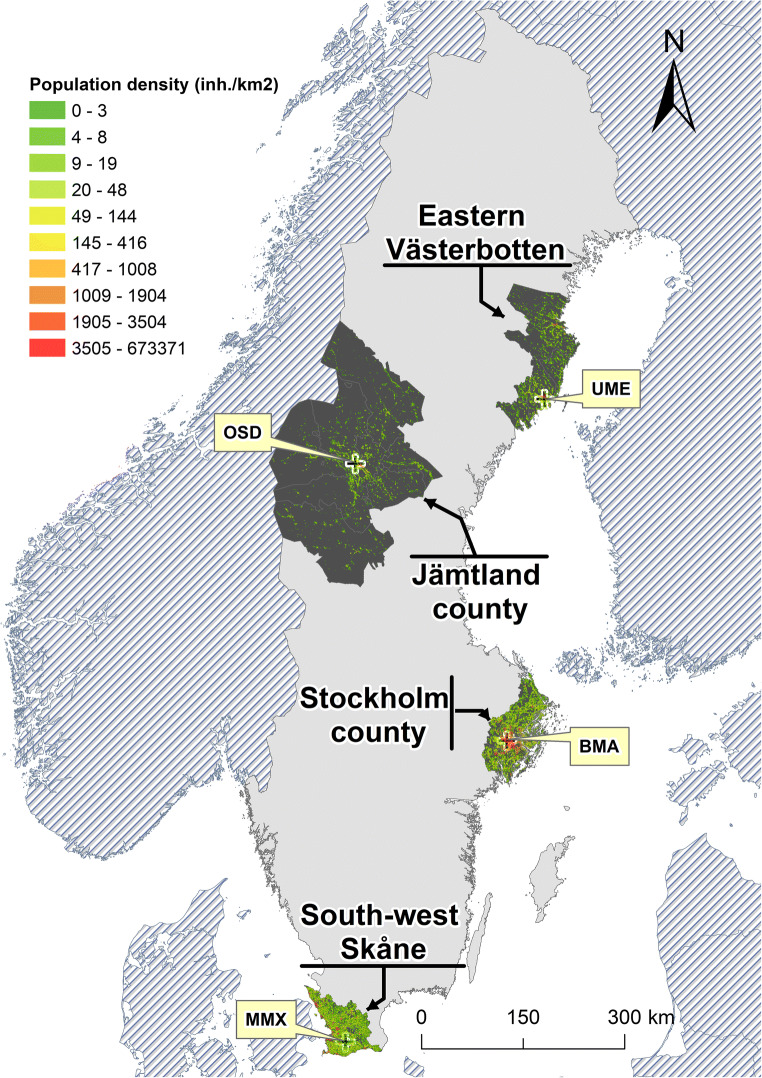
The four study locations in Sweden. Locations of main weather stations in each study area are represented by black crosses (Malmö—MMX, Bromma—BMA, Östersund—OSD and Umeå—UME). Population density (inhabitants/km^2^) was divided into deciles. Population data source: ©SCB (Statistics Sweden)

The study was performed in the following four Swedish locations: south-west Skåne (Scania) county (21 municipalities), Stockholm county (26 municipalities), Jämtland county (12 municipalities) and eastern Västerbotten county (5 municipalities) (Fig. 1). The population data represented in the map is from 2014, the last year of the study period. It was obtained from ©SCB (Statistics Sweden) (SCB—Centralbyrån Statistiska 2014).

For each of these regions, daily mortality totals were calculated, where deaths are grouped according to area of residence. The selection of cases by specific causes of death was based on the International Statistical Classification of Diseases and Related Health Problems (WHO 1978, 2016). ICD-9 codes were used from 1991 to 1998 and ICD-10 codes from 1999 to 2014. Daily deaths by cardiovascular diseases (ICD-9 390–459, ICD-10 I00–I99) and by respiratory diseases (ICD-9 460–519, ICD-10 J00–J99) from January 1, 1991, to December 31, 2014, were obtained from the Linnaeus database at the Centre for Demographic and Ageing Research (CEDAR), Umeå University, Sweden. This database provides national, health-related, demographic and lifestyle variables for the Swedish population (Malmberg et al. 2010).

Meteorological measurements every 6 hours each day were obtained from the Swedish Meteorological and Hydrological Institute (SMHI). Specifically, data were obtained from the following weather stations: Malmö A (Skåne), Bromma airport (Stockholm), Östersund (Jämtland) and Umeå airport (Västerbotten). Where data were missing, to improve the completeness of data, meteorological observations were taken from adjacent stations located no more than 100 km from the main weather station but inside the corresponding study location, and for missing hourly data, interpolation was done from adjacent hours. Overall, we replaced under 2% of observations at each station, with under 1% at Stockholm. The SSC requires 24 observations per day (6 variables for each of 4 time periods); for nearly all days where observations were not complete, the number of missing observations was 4 or fewer, and thus, the spatial and temporal interpolation had minimal impact on the characterisation of weather of the day. The geographic location of the weather stations is shown in Fig. 1. Characteristics of weather types per location and month are shown in supplementary Table S1.

### Statistical analysis

For the study, the full period was divided into the following two seasons: extended summers (May–September; hereafter, “summer”) and extended winters (November–March, hereafter, “winter”), each assessed separately. During the summer, we explored the lag structure and cumulative effect over 14 days of hot weather types (DT and MT) on mortality, by cardiovascular and respiratory diseases. Additionally, for estimating the association between cardiovascular and respiratory mortality and heat events, hot days were defined as either DT or MT weather type, based on their comparable health effect estimated in previous studies (Fonseca-Rodríguez et al. 2019; Sheridan and Kalkstein 2004; Sheridan and Lin 2014). The length of the heat events was determined by the number of consecutive hot days (either DT or MT) (Sheridan and Lin 2014); analysis in which consecutive days of DT and MT individually was also explored, but as many heat events in Sweden are of mixed DT and MT types over multiple days, the sample sizes for events that are entirely one type only are minimal. The effect of each day in sequence of a heat event on cardiovascular and respiratory mortality at lag 0 and the cumulative effect over 14 days were estimated. Thus, we included 1–7 hot days in sequence (DIS) in summer, with the days that were not part of heat events serving as the reference category.

Similarly, in winter we assessed the lag structure and cumulative effect over 28 days of the coldest weather types (DP and MP) on cause-specific mortality. Furthermore, we analysed the effect of the duration of cold events determined by cold days (either DP or MP) in sequence, estimating the effect at lag 0 and the cumulative effect over 28 days on cardiovascular and respiratory mortality during winter. In winter, we included up to 10 cold DIS to study the duration of cold events. Days that were not part of cold events were considered the reference category.

A time-stratified case-crossover design was carried out to evaluate the relationship between the outcome and the independent variables. Time-stratified case-crossover analysis and Poisson regression time-series analysis produce quantitatively similar results (Basu et al. 2005). We used conditional Poisson regression with stratum indicator variable conditioning on numbers of events in each time stratum (Armstrong et al. 2014; Levy et al. 2001). A stratum variable was composed for year, month and day of the week, allowing us to control for long-term trends and seasonal and weekday effects, assuming that unmeasured time-dependent confounders are constant within a stratum (Lu et al. 2008). Thus, the case and control days were compared within the same stratum; hence, each case day was matched to its control days on the same day of the week in the same month and the same year (Kim et al. 2019).

The conditional Poisson regression is a flexible alternative to the traditional conditional logistic regression used in case-crossover designs (Armstrong et al. 2014). This approach allows researchers to control for autocorrelation and overdispersion of time series data more effectively than conditional logistic regression. It also offers results equivalent to those obtained by using a conditional logistic regression when there is a common exposure across individuals (Armstrong et al. 2014; Oudin Astrom et al. 2018). Moreover, the conditional Poisson regression has an important advantage over the unconditional Poisson regression, also used in this type of studies, because the conditional Poisson regression reduces the number of estimated parameters and the computation time without affecting the accuracy of the parameter estimation (Armstrong et al. 2014).

All statistical analyses were performed using R statistical software (R Development Core Team 2018). The conditional Poisson regression model was extended to a quasi-Poisson variant, to account for overdispersion, by fitting an extra dispersion parameter. It was carried out using the *gnm* package. The distributed lag relationship between weather types and heat waves and mortality by cardiovascular and respiratory diseases (Gasparrini et al. 2010) was assessed using the *dlnm* package. Distributed lag nonlinear models (DLNMs) use a cross-basis function to represent the delayed nonlinear exposure–outcome relationships (Gasparrini 2011). In our analysis, we assessed the lag structure of the effects and the cumulative impact of weather types on mortality over 14 days in summer and 28 days in winter, with knot placements for the lags at three equally spaced positions. The *logknots* function (from the *dlnm* package) defines knots for lag space at equally spaced log-values, and it was expressly created for lag–response functions (Gasparrini et al. 2019).

The model used in our study was as follows:

$$ {Y}_t\sim quasi- Poisson\left({\mu}_t,\theta \right) $$

$$ \mathrm{Var}\ \left({Y}_t\right)=\theta \mu $$

$$ Log\left({\mu}_t\right)=\alpha +\beta Crossbasis\left( Weather\ types\right)+{\lambda Stratum}_t+ Offset\ \left(\mathit{\log}\  of\ Population\right) $$

The model outcome was daily deaths (*Y*_t_) by cardiovascular and respiratory diseases. *α* is the intercept. Cross-basis matrix of binary variables (*Weather types*) were created for each of the weather types considered in summer (dry tropical—DT and moist tropical—MT) and in winter (dry polar—DP and moist polar—MP). Additionally, to study the effect of the duration of heat events and cold events, binary variables were created for each DIS, from 1 to 7 hot days in summer and from 1 to 10 cold days in winter. During the study period, the percentages of hot DIS are longer than 7 days (8th and beyond) in summer and cold DIS are longer than 10 days (11th and beyond) in winter were less than 2% and 3%, respectively. The small number of hot DIS higher than 7 and cold DIS higher than 10 highly affected the precision of the estimation producing very wide confidence intervals and were not considered in the analysis. The stratum is an indicator variable composed of year, month and day of the week (year, month, DOW). The parameters of the stratum are not estimated and are “conditioning out” by conditioning on the number of deaths in each time stratum (Armstrong et al. 2014). The count of the total population was interpolated linearly to the daily level by annual population counts for each location. The daily population under risk was used as the offset variable.

We computed relative risks (RRs) of mortality with 95% confidence intervals (CIs) for each weather type. Also, RR at lag 0 and cumulative RR at lag 14 were estimated for hot DIS, and RR at lag 0 and cumulative RR at lag 28 were estimated for cold DIS. We used a 14-day maximum lag in summer because the effect of heat is typically immediate, and 14 days is sufficiently long to capture the acute effects of weather (Braga et al. 2001; Fonseca-Rodríguez et al. 2019; Sheridan and Lin 2014). In winter, we used a 28-day lag because cold weather usually produces a delayed effect up to 25 days according to Analitis et al. (2008) and Anderson and Bell (2009).

As a sensitivity analysis, we estimated the cumulative effect of DT and MT over 14 days in summer and DP and MP over 28 days in winter for both outcomes using conditional Poisson regression (CPR) and Poisson regression time-series analysis (TSA). In general, the results of CPR and TSA are similar; however, the CPR showed wider confidence intervals. The compared models and their results are shown in the Supplementary Material (Fig. S5 and Fig. S6).

## Results

Cardiovascular and respiratory mortality rates varied by location and month. The highest monthly mean mortality rate for cardiovascular diseases was observed in Jämtland, ranging from 42.0 per 100,000 inhabitants in July to 56.2 in January, while the lowest rate was observed in Stockholm (ranging from 25.6 in August to 31.8 in January) (Supplementary Material, Fig. S2**)**. Similarly, Jämtland showed the highest monthly mean respiratory mortality rate, with a peak in January (11.8) and the lowest rate (5.8) in July. Stockholm and Västerbotten had the lowest mean mortality rate of the four study locations (Fig. S4).

The time series of daily deaths and monthly mortality rates by cardiovascular (Fig. S1) and respiratory diseases (Fig. S3) as well as characteristics of each weather type (Table S1) for January and July, and frequency of hot and cold DIS (Table S2) in summer and winter, respectively, at each location are shown in the Supplementary Material.

The daily cardiovascular and respiratory disease mortality rates in summer were generally higher on DT days than on MT days and were also higher compared with the days with not hot weather types. In winter, days with DP weather also exhibited the highest cardiovascular and respiratory mortality rates in northern and southern locations (Table 1).

**Table 1:** Descriptive statistics of cardiovascular and respiratory diseases and weather types in the four study locations, 1991–2014

Location		Summer			Winter		
		Not hot WT	DT	MT	Not cold WT	DP	MP
Skåne	Percentage of days	83.25	8.28	8.47	67.11	7.52	25.37
	Cardiovascular mortality	1.04 (1.03–1.05)	1.11 (1.07–1.15)	1.03 (0.99–1.06)	1.21 (1.2–1.23)	1.25 (1.21–1.3)	1.24 (1.22–1.27)
	Respiratory mortality	0.17 (0.16–0.17)	0.19 (0.18–0.21)	0.19 (0.18–0.21)	0.24 (0.23–0.24)	0.25 (0.23–0.27)	0.24 (0.23–0.26)
Stockholm	Percentage of days	80.11	10.12	9.77	52.44	16.53	31.03
	Cardiovascular mortality	0.85 (0.84–0.86)	0.89 (0.87–0.91)	0.84 (0.82–0.86)	0.98 (0.97–0.99)	1.01 (0.99–1.03)	0.99 (0.97–1)
	Respiratory mortality	0.13 (0.13–0.14)	0.15 (0.14–0.16)	0.14 (0.13–0.15)	0.18 (0.18–0.19)	0.19 (0.18–0.2)	0.18 (0.17–0.18)
Jämtland	Percentage of days	87.16	5.94	6.9	60.31	7.94	31.75
	Cardiovascular mortality	1.48 (1.44–1.52)	1.42 (1.29–1.57)	1.31 (1.19–1.44)	1.69 (1.64–1.73)	1.72 (1.59–1.86)	1.69 (1.62–1.75)
	Respiratory mortality	0.21 (0.2–0.22)	0.2 (0.16–0.26)	0.23 (0.18–0.29)	0.32 (0.3–0.34)	0.34 (0.28–0.4)	0.3 (0.27–0.33)
Västerbotten	Percentage of days	86.64	5	8.36	50.47	22.44	27.09
	Cardiovascular mortality	1.01 (0.98–1.03)	1.06 (0.96–1.17)	0.87 (0.8–0.95)	1.15 (1.12–1.19)	1.26 (1.21–1.32)	1.1 (1.06–1.15)
	Respiratory mortality	0.12 (0.11–0.13)	0.1 (0.07–0.14)	0.14 (0.11–0.17)	0.18 (0.17–0.19)	0.2 (0.18–0.22)	0.17 (0.15–0.19)

*DT*, dry tropical; *MT*, moist tropical; *DP*, dry polar; *MP*, moist polar; *WT*, weather type; *Mortality*, daily mortality rate per 100,000 inhabitants with 95% CI

### Summer

**Fig. 2:**
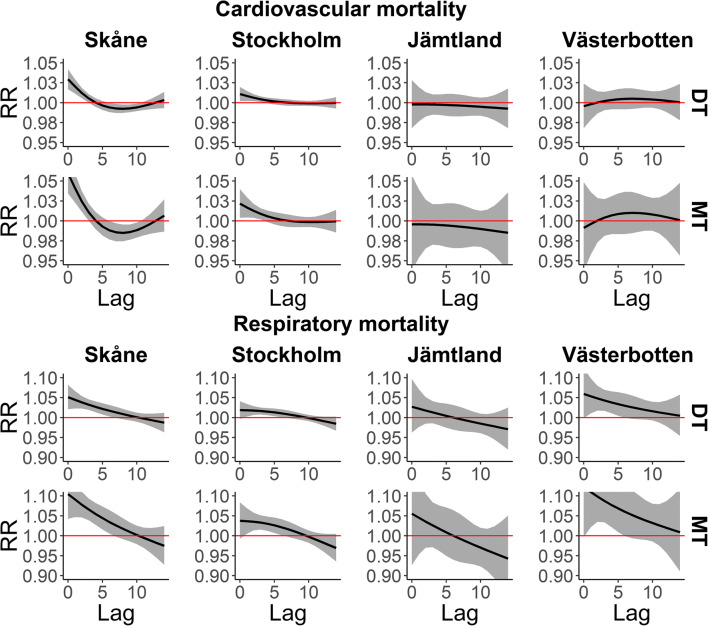
Mortality (lag-distributed RR with 95% CI) by cardiovascular (top) and respiratory (bottom) diseases related to hot (DT and MT) oppressive weather types during summer (May–September) in the four study areas

In summer, the DT and MT weather increased the mortality risk for cardiovascular diseases at shorter lags in Skåne and in Stockholm, showing a large and clear effect in increasing the cardiovascular mortality until lag 3 in both locations. However, the RR did not increase in northern locations. Interestingly, in Skåne, the MT showed a clear reduction of cardiovascular mortality between lag 6 and lag 10 (Fig. 2).

The effect of DT and MT weather on respiratory disease mortality in summer is shown in Fig. 2. Both hot weather types (DT and MT) were associated with an increase of mortality risk until lag 6 in the two southern locations (Skåne, Stockholm) and in the northernmost location (Västerbotten). Interestingly, the largest effect of MT on respiratory mortality occurred in Västerbotten.

**Fig. 3:**
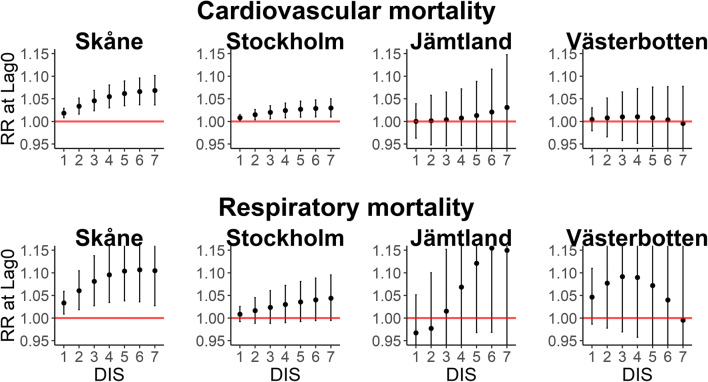
Zero-day lag RRs and 95% CI of heat events (DT and MT) in sequence during summer and mortality by cardiovascular (top) and respiratory (bottom) diseases. DIS = days in sequence

In general, the impact of heat events on mortality by cardiovascular and respiratory diseases increased from day 1 to day 7 but not in Västerbotten (Fig. 3). The RR of mortality by cardiovascular diseases at lag 0 was large (DIS 1–7) in the two southern locations but small in the northern ones with wide CIs. Also, mortality by respiratory diseases increased from day 1 to day 7 of the heat event in Skåne, Stockholm and Jämtland. Despite large RR in Jämtland and Västerbotten, the precision was very low, reflected by wide CIs.

The persistence of DT and MT weather types over multiple days did not show an increase of cumulative risk of cardiovascular disease mortality (Table 2) overall. In Stockholm, the cumulative RR of cardiovascular mortality increased almost linearly from day 1 to day 7 of the heat event associated with the tropical weather conditions, and this association was more reliable at day 7.

**Table 2:** Cumulative RR and 95% CI over 14 days of hot days (DT and MT) and heat events (hot days in sequence)

		Cardiovascular mortality			
		Skåne	Stockholm	Jämtland	Västerbotten
Hot days	DT	1.03 (0.97–1.09)	1.03 (0.99–1.07)	0.94 (0.82–1.09)	1.04 (0.91–1.17)
	MT	1.06 (0.95–1.18)	1.07 (0.99–1.15)	0.89 (0.66–1.18)	1.07 (0.83–1.38)
Heat events (hot DIS)	1	1.00 (0.94–1.06)	1.01 (0.97–1.05)	1.04 (0.81–1.35)	0.89 (0.77–1.03)
	2	1.00 (0.91–1.10)	1.02 (0.96–1.08)	1.00 (0.71–1.42)	0.85 (0.68–1.07)
	3	1.01 (0.90–1.13)	1.03 (0.96–1.11)	0.91 (0.65–1.28)	0.86 (0.66–1.14)
	4	1.01 (0.89–1.15)	1.05 (0.96–1.14)	0.80 (0.58–1.10)	0.92 (0.69–1.23)
	5	1.02 (0.90–1.16)	1.06 (0.98–1.16)	0.70 (0.48–1.02)	1.02 (0.76–1.38)
	6	1.03 (0.91–1.17)	1.08 (0.99–1.18)	0.63 (0.38–1.04)	1.17 (0.85–1.61)
	7	1.04 (0.91–1.19)	1.10 (1.01–1.21)	0.59 (0.32–1.12)	1.36 (0.94–1.98)
		Respiratory mortality			
		Skåne	Stockholm	Jämtland	Västerbotten
Hot days	DT	1.25 (1.09–1.43)	1.09 (0.99–1.19)	0.96 (0.71–1.30)	1.51 (1.14–2.01)
	MT	1.57 (1.20–2.05)	1.18 (0.98–1.43)	0.92 (0.50–1.69)	2.29 (1.30–4.04)
Heat events (Hot DIS)	1	1.06 (0.92–1.22)	1.03 (0.94–1.14)	0.59 (0.33–1.05)	1.41 (1.00–1.99)
	2	1.13 (0.89–1.42)	1.07 (0.91–1.25)	0.50 (0.23–1.10)	1.86 (1.08–3.20)
	3	1.21 (0.91–1.61)	1.09 (0.90–1.33)	0.56 (0.26–1.20)	2.31 (1.24–4.30)
	4	1.30 (0.96–1.78)	1.12 (0.90–1.38)	0.75 (0.38–1.48)	2.68 (1.40–5.12)
	5	1.41 (1.03–1.92)	1.13 (0.91–1.40)	1.12 (0.53–2.38)	2.92 (1.46–5.82)
	6	1.52 (1.11–2.07)	1.15 (0.92–1.42)	1.69 (0.62–4.64)	2.98 (1.31–6.77)
	7	1.63 (1.18–2.24)	1.15 (0.92–1.45)	2.38 (0.66–8.55)	2.86 (1.00–8.16)

*DT*, dry tropical; *MT*, moist tropical; *DIS*, days in sequence

A high cumulative impact of DT and MT on mortality by respiratory diseases was identified in Skåne and Västerbotten. Also, the cumulative effect of hot DIS from day 5 to 7 and day 2 to 7 in Skåne and Västerbotten, respectively, was clearly high, and additionally in those locations, the cumulative RR of DIS showed an increasing trend over the duration of the heat events (Table 2).

### Winter

**Fig. 4:**
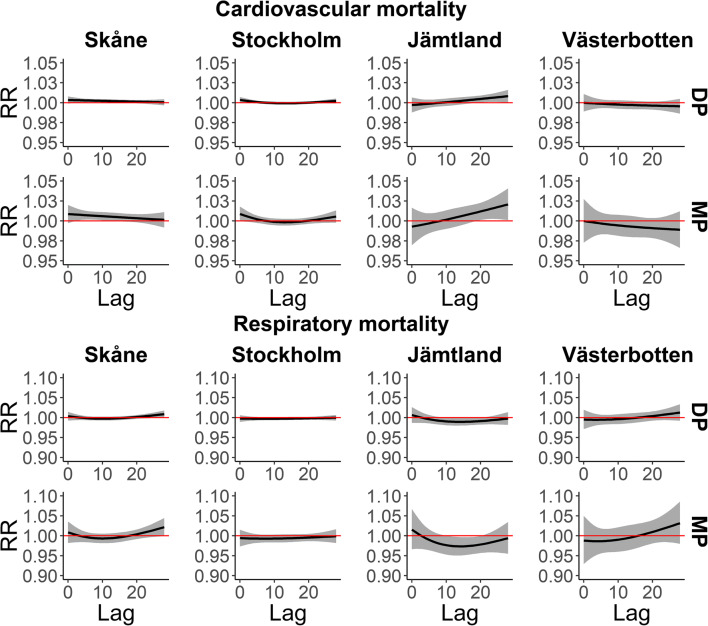
Mortality (lag-distributed RR with 95% CI) by cardiovascular (top) and respiratory (bottom) diseases related to cold (DP and MP) oppressive weather types during winter (November–March) in the four study areas

The DP weather type showed a negligible effect on mortality for cardiovascular and respiratory diseases in the study locations. However, the MP weather type slightly increased the mortality risk for cardiovascular diseases from lag 3 to lag 13 in Skåne, but the effect was more immediate in Stockholm, where the mortality risk increased from lag 0 to lag 3. This effect was more delayed in Jämtland, showing a substantial mortality increase from lag 19 to lag 28 (Fig. 4).

The effect of DP and MP cold weather types was even lower for mortality by respiratory diseases than by cardiovascular diseases (Fig. 4). Interestingly, in Jämtland the MP weather showed a reduction of RR from lag 11 to lag 20.

**Fig. 5:**
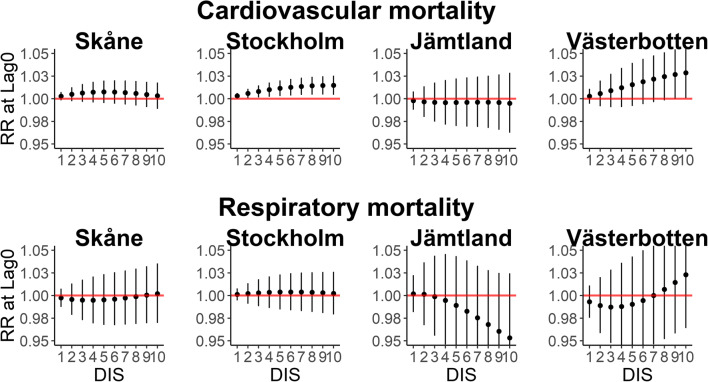
Zero-day lag RRs and 95% CI of cold day (DP and MP) in sequence during winter and mortality by cardiovascular (top) and respiratory (bottom) diseases. DIS = days in sequence

The duration (DIS) of cold events at lag 0 was not associated with increased mortality for cardiovascular diseases in Skåne and Jämtland, but it was more present in Stockholm and Västerbotten, where the effect of cold DIS increased more or less linearly from day 1 to day 10. On the other hand, the cold events did not seem to produce a consistent effect on mortality by respiratory diseases at any of the sites (Fig. 5). In Jämtland, cold DIS appeared to reduce the respiratory mortality; however, the imprecision of the estimate was considerable.

The cumulative effect of the DP and MP weather as well as cold events (cold DIS from day 1 to day 10) over 28 days (lag 28) on mortality by cardiovascular disease was large in Skåne, the southernmost location. This effect was not clear in the rest of the study locations. Nonetheless, in Stockholm and Jämtland, the longer the duration of the cold event, the higher the cumulative RR of cardiovascular mortality, although the effect was not conclusive because of broad CIs.

The DP and MP weather did not produce an important cumulative impact on respiratory mortality over 28 days. Also, no evident cumulative effect of cold DIS on mortality by respiratory disease was identified in the four study locations. However, in southern locations the RR increased across the DIS, but in Jämtland, it seems to have decreased over the duration of cold events (Table 3).

**Table 3:** Cumulative RR and 95% CI over 28 days of cold days (DP and MP) and cold events (cold days in sequence)

		Cardiovascular mortality			
		Skåne	Stockholm	Jämtland	Västerbotten
Cold days	DP	1.06 (1.01–1.11)	1.02 (0.98–1.06)	1.07 (0.96–1.20)	0.93 (0.82–1.05)
	MP	1.15 (1.02–1.31)	1.05 (0.95–1.15)	1.19 (0.90–1.56)	0.83 (0.61–1.12)
Cold events (cold DIS)	1	1.04 (1.01–1.08)	1.00 (0.96–1.04)	0.98 (0.85–1.13)	0.94 (0.84–1.05)
	2	1.08 (1.02–1.14)	1.01 (0.94–1.08)	0.98 (0.77–1.24)	0.89 (0.73–1.09)
	3	1.11 (1.03–1.19)	1.01 (0.92–1.11)	0.98 (0.73–1.33)	0.87 (0.67–1.13)
	4	1.13 (1.04–1.23)	1.02 (0.92–1.14)	1.00 (0.71–1.39)	0.86 (0.64–1.16)
	5	1.14 (1.04–1.25)	1.03 (0.92–1.16)	1.02 (0.72–1.44)	0.86 (0.63–1.18)
	6	1.15 (1.05–1.26)	1.05 (0.93–1.18)	1.04 (0.74–1.47)	0.87 (0.63–1.21)
	7	1.15 (1.04–1.26)	1.06 (0.94–1.20)	1.08 (0.77–1.50)	0.90 (0.65–1.24)
	8	1.14 (1.04–1.25)	1.08 (0.96–1.22)	1.11 (0.80–1.54)	0.93 (0.68–1.28)
	9	1.13 (1.03–1.24)	1.10 (0.98–1.23)	1.15 (0.82–1.61)	0.98 (0.72–1.33)
	10	1.12 (1.01–1.23)	1.12 (0.99–1.25)	1.18 (0.82–1.71)	1.03 (0.75–1.41)
		Respiratory mortality			
		Skåne	Stockholm	Jämtland	Västerbotten
Cold days	DP	1.03 (0.91–1.16)	0.94 (0.86–1.03)	0.84 (0.66–1.05)	1.01 (0.76–1.35)
	MP	1.07 (0.79–1.45)	0.86 (0.68–1.08)	0.64 (0.36–1.13)	1.03 (0.50–2.11)
Cold events (cold DIS)	1	1.00 (0.87–1.16)	0.98 (0.89–1.07)	1.04 (0.77–1.40)	1.11 (0.86–1.45)
	2	1.02 (0.80–1.30)	0.97 (0.82–1.14)	1.04 (0.63–1.73)	1.20 (0.76–1.90)
	3	1.03 (0.76–1.42)	0.96 (0.77–1.19)	1.01 (0.53–1.91)	1.26 (0.70–2.28)
	4	1.06 (0.74–1.51)	0.96 (0.75–1.24)	0.95 (0.47–1.92)	1.28 (0.65–2.53)
	5	1.08 (0.74–1.58)	0.97 (0.74–1.27)	0.87 (0.42–1.80)	1.27 (0.62–2.63)
	6	1.11 (0.76–1.64)	0.98 (0.74–1.30)	0.79 (0.38–1.61)	1.24 (0.59–2.58)
	7	1.15 (0.78–1.68)	1.00 (0.75–1.32)	0.70 (0.35–1.40)	1.18 (0.57–2.44)
	8	1.18 (0.81–1.71)	1.02 (0.77–1.35)	0.61 (0.30–1.22)	1.11 (0.54–2.26)
	9	1.21 (0.83–1.76)	1.04 (0.79–1.37)	0.53 (0.26–1.10)	1.02 (0.50–2.08)
	10	1.23 (0.84–1.82)	1.06 (0.81–1.40)	0.46 (0.21–1.02)	0.93 (0.45–1.94)

*DP*, dry polar; *MP*, moist polar; *DIS*, days in sequence

## Discussion

This study is novel in that relatively few studies have compared summer and winter weather impacts directly, and very few have explored smaller metropolitan areas in subarctic climates. Specifically, we studied cause-specific mortality during summer in relation to dry tropical and moist tropical weather, and in relation to dry or moist tropical heat events stretching over a duration of up to 7 days. We also studied the association of cause-specific mortality with dry polar and moist polar weather during winter and the effect of the duration of cold days in sequence (cold events) up to 10 days.

### Summer

The hot weather types (DT and MT) had an immediate and adverse effect on mortality by cardiovascular diseases in southern locations, this effect being larger in Skåne than in Stockholm. In Skåne, we observed a clear reduction in mortality rates around 5 days after the exposure to both hot weather types (DT and MT), lasting for 4–5 days. A similar effect produced by high temperatures on mortality has been shown in previous studies in Sweden (Rocklöv and Forsberg 2008; Rocklöv and Forsberg 2010) and other geographic areas (Baccini et al. 2013; Hajat et al. 2005). The populations in northern locations (Jämtland and Västerbotten) did not appear to be affected by dry or moist tropical weather in terms of cardiovascular mortality. In our previous study (Fonseca-Rodríguez et al. 2019), we found that all-cause mortality was increased by DT and MT during summer in southern locations, showing a pattern similar to cardiovascular mortality in the present study. This could be explained by the high proportion of deaths by cardiovascular causes that are around 40% of total deaths in our study region. Sheridan and Lin (2014) found a strong heat-health relationship between hot days (DT or MT) and all-cause mortality and cardiovascular mortality. Additionally, in most of the 44 cities in a study from the USA, both weather types were associated with excess mortality, and MT had a higher impact than did DT (Kalkstein and Greene 1997).

In a multi-city study, in northern Europe, no significant increase in cardiovascular mortality due to heat was detected (Baccini et al. 2008). Although smaller in scale, our study identified a different impact of hot weather on cardiovascular mortality in Sweden, showing a clear high effect in southern locations and not in the northern ones. Also, Rocklöv et al. (2011) found an elevated death rate for cardiovascular diseases associated with heat in Stockholm. Additionally, high temperatures have been associated with mortality in people younger than 65 years and affected by myocardial infarction (Rocklöv et al. 2014).

In a study conducted in Prague, Czech Republic, DT and MT weather types were associated with an excess of cardiovascular mortality; in addition, the authors found that the highest impact was produced by DT (Urban and Kysely 2018). Conversely, our study showed a higher impact of MT than of DT. This is in line with the results obtained by Zeng et al. (2017), showing that the impact of temperature on cardiovascular death was higher at high humidity. The temperature on DT days is higher than on MT days; however, the latter clearly have higher humidity, and this characteristic could increase the impact on mortality. The high humidity can reduce the body’s efficiency of evaporative heat loss through perspiration (Ding et al. 2016), exacerbating the temperature effects on people with cardiovascular problems (Zeng et al. 2017). Armstrong et al. (2019), on the other hand, found that humidity does not increase the mortality risk over 3 days during summer once temperature was accounted for. While this contrasts with expectations from previous physiological studies (Davis et al. 2016; McGregor and Vanos 2018), it is consistent with other epidemiological studies (Barnett et al. 2010) in which heat indices including humidity produced little evidence of improving predictions.

The risk of cardiovascular mortality increased over the duration of heat events, mostly in southern locations, where those events are also more frequent and hotter than in northern locations. This could be because the longer the heat event, the greater the burden on the cardiovascular system (Barnett et al. 2012). Other studies have shown an association between the duration of heat events and increased cardiovascular mortality (Hajat et al. 2002; Linares et al. 2015). However, in a recent study (Urban and Kysely 2018) in the Czech Republic, the persistence of hot (DT and MT) oppressive weather conditions was not associated with cardiovascular mortality. In a previous study also conducted in Sweden, the authors found that heat wave duration was associated with increased mortality in people older than 65 years with pre-existing cardiovascular disease (Rocklöv et al. 2014). Although there was no stratification by age in our study, most deaths occurred in the population older than 65.

Respiratory mortality was also associated with hot weather in our study. Although the pathophysiological mechanisms remain unclear (Basu and Samet 2002), previous studies have noted an increase of respiratory mortality during heat events (Basu 2009; Braga et al. 2002). Also in Stockholm, Rocklöv et al. (2014) showed an association between high temperature and mortality in the elderly population with a pre-existing respiratory disease. In addition, when temperature increased, mortality increased in people with chronic obstructive pulmonary disease (COPD) (Rocklöv et al. 2014). COPD is one of the main causes of chronic morbidity and also mortality in Sweden (Lisspers et al. 2018; Ställberg et al. 2014), and studies have shown that high temperatures combined with high humidity cause COPD symptoms to worsen (De Pietro 2018). In our study, moist tropical (MT) weather produced the largest effect on respiratory mortality compared with all other weather types, hinting at COPD patients being a high-risk group during moist hot weather in southern (Skåne and Stockholm) and northern (Västerbotten) regions of Sweden. Air pollution could also play a role in respiratory mortality, increasing during MT and DT days because during hot days, levels of air pollution (e.g. CO and NO_2_) are higher than during other weather types (Vanos et al. 2014).

Interestingly, in the present study, hot weather had a higher effect on respiratory mortality than on cardiovascular mortality, in line with previous studies (Baccini et al. 2008; Hajat et al. 2002). Likewise, Vanos et al. (2014) found that respiratory mortality is higher than cardiovascular mortality due to air pollution on DT and MT days.

Other studies have noted the relationship between the duration of a heat event and increasing respiratory mortality, showing that the longer the duration of a heat wave, the greater its impact on mortality (Anderson and Bell 2011; Hajat et al. 2002; Linares et al. 2015). A significant increase in daily heat-related mortality has been observed for total, respiratory and other cause-specific deaths in several European cities. Also, an association with heat wave duration was found (D’Ippoliti et al. 2010).

In humans, excessive heat increases the respiratory rate, causing thermal hyperpnea (White 2006). This increased pulmonary ventilation that worsens the effects of chronic obstructive pulmonary disease (Anderson et al. 2013) due to persistent pulmonary and systemic inflammation and ventilatory impairment (Mannino et al. 2006). Also, people with COPD have increased risk of developing cardiovascular complications causing mortality (Mannino et al. 2006; Sin Don and Man 2003).

### Winter

The dry polar (DP) and moist polar (MP) weather types increased the cardiovascular mortality in southern locations and in Jämtland (north), but in general, the increase was very slight and almost negligible in southern locations compared with the effect of hot weather. Similarly, Kalkstein and Greene (1997) found that DP and MP increased mortality slightly in certain US cities during the winter, but this effect was considerably smaller than the excess deaths associated with MT and DT air in summer. Lee (2015b) showed an association between cold weather and cardiovascular mortality in a multi-city study, but conversely, he found that dry and cold weather had a higher impact than more humid cold weather types. Huynen and Martens (2015) also found a lower effect of cold than heat exposure on cardiovascular mortality in a study conducted in the Netherlands. Similarly, other authors have demonstrated the association between cold weather and cardiovascular mortality in different geographic areas (Han et al. 2017; Murage et al. 2018).

The cold weather (DP and MP) and cold events (DIS) showed a slight or unclear effect on cardiovascular and respiratory mortality. However, the effect of cold weather was higher on cardiovascular mortality compared to respiratory mortality. Likewise, (Hajat et al. 2007) found that in England and Wales cardiovascular mortality was higher than respiratory mortality due to cold temperatures. Our findings show that the duration of cold events was associated with an increase in cardiovascular mortality in Stockholm and Västerbotten at a 0-day lag. In Skåne we also found a cumulative effect of a 28-day cold DIS duration on cardiovascular mortality. The duration of cold events is a risk factor for an increase in cardiovascular mortality, as reported by other authors (Wang et al. 2016). Also, a recent study found that a long duration of extreme cold events generally results in a much larger and significantly increased rate of mortality (Smith and Sheridan 2018). However, in a previous study conducted in Stockholm, Sweden, no association was found between duration of cold spells and cardiovascular mortality (Rocklöv et al. 2011; Rocklöv et al. 2014).

We did not find a substantial effect of cold weather (DP and MP) on respiratory mortality, consistent with other studies from Sweden (Rocklöv et al. 2011; Rocklöv et al. 2014) or the U.S. (Braga et al. 2002). Nonetheless, previous studies have suggested that low temperatures increase the risk of death by respiratory diseases; these studies have been conducted in geographic areas including England, UK (Murage et al. 2018), China (Han et al. 2017), Spain (Linares et al. 2015), and 15 European cities (Analitis et al. 2008). In addition, in the present study the duration of cold events did not show a relevant impact on respiratory mortality, in line with two previous Swedish studies (Rocklöv et al. 2011; Rocklöv et al. 2014). Different results, however, were found by Wang et al. (2016), who reported that the duration of cold events significantly increases respiratory mortality.

The higher impact of heat compared to cold on cause-specific mortality in Sweden could be because the Swedish population is better adapted to cold weather, and because housing conditions with good insulation and warming systems protect residents from cold weather. However, insulation conditions might be insufficient during hot days. Behavioural and physiological adaptation reduces the harmful effect of weather conditions, in particular of high temperatures; thus, heat-related deaths are more common in areas in which excessive heat is rare (Kinney et al. 2008). This appears to be the case even in the cold climate of our study locations.

In general, studies before showed the impacts of high temperature with a focus on mid-latitude locations. In our study, however we could show with the SSC that is important whether the weather is dry or humid, considering what is typical for a location at a given time of the year. It makes the SSC a well-suited system that endeavour to capture these synergistic effects on human health (Hondula et al. 2014). The SSC approach identifies hot and cold days based on season-specific thresholds and applies a holistic assessment of the day; thus, it results in a different identification of heat events than other methods. Therefore, comparability to studies using a different approach of heat and cold events is limited.

The method of conditional Poisson regression used in this study is equivalent to the time-stratified case-crossover analysis using conditional logistic regression and to the time-series Poisson regression analyses which reportedly produce comparable results (Armstrong et al. 2014; Basu et al. 2005; Lu and Zeger 2006). The conditional Poisson model is an alternative to the traditional conditional logistic model, offering some advantages such as allowing for overdispersion, autocorrelation in the original counts and for varying rate denominators. Also, the programming is simpler and computationally less intensive than conditional logistic model (Armstrong et al. 2014).

However, the present study has several limitations that should be acknowledged. First, we did not examine the potentially confounding effect of air pollution. The concentration of some pollutants such as NO2, SO2, CO and O3 in the atmosphere varies across different weather types and seasons, and cardiovascular and respiratory mortality is associated with those pollutants (Vanos et al. 2014). Also, particulate matter (PM10 and PM2.5) was not considered in this study. The absence of adjustment for influenza incidence, which is associated with mortality as well as with weather conditions, could be another potential limitation of this study. However, by controlling for seasonality and trend, we can capture much of seasonal influenza epidemics effects. Additionally, the small number of daily deaths in northern locations (Jämtland and Västerbotten) reduced the precision of the estimates.

## Conclusions

The effect of hot and cold oppressive weather and of the duration of heat and cold events on mortality by cardiovascular and respiratory diseases was assessed during summer and winter in southern and northern locations in Sweden. In general, the present study shows that hot weather types have a greater impact on cause-specific mortality than do cold weather types. Also, heat affected cardiovascular and respiratory mortality more in southern than in northern locations. Moist tropical (MT) weather had a higher impact than did dry tropical (DT) weather on cardiovascular and respiratory mortality. Furthermore, heat had a higher impact on respiratory mortality than on cardiovascular mortality. The duration of hot events (DIS) was associated with cardiovascular mortality and respiratory mortality, showing a clearer effect in southern locations.

This study provides valuable information about the relationship between hot oppressive weather types and cause-specific mortality. It could contribute to the adoption of preventive measures to reduce the mortality risk. The results obtained in the present study will be useful in projecting the future impact of oppressive weather types on morbidity and mortality, based on climate and demographic change. Thus, information about harmful weather conditions beyond high and low temperatures must be communicated to stakeholders and health staff in homes for the elderly, hospitals, general practitioners etc. about health risks related to specific weather types for vulnerable patients. Particular attention should be paid for people with chronic cardiovascular and respiratory diseases.

The cold weather types (MP and DP) may not capture sufficiently effects of cold extremes on mortality in this sub-Arctic region during winter, given how common these types are at that time of year. Thus, we recommend considering a new approach, perhaps separating “extreme cold” weather types (subsets of dry polar and moist polar) from the current polar types to assess impacts of cold weather on health outcomes.

## Electronic supplementary material

ESM 1 (DOCX 1536 kb)
