# Supplementary material for: Hot and cold weather based on the spatial synoptic classification and cause-specific mortality in Sweden: a time-stratified case-crossover study
Source: Int J Biometeorol. 2020 Apr 23;64(9):1435–49. doi: 10.1007/s00484-020-01921-0 (PMC7445203; doi:10.1007/s00484-020-01921-0)
Supplement: Supplementary file 1 — (DOCX 1536 kb) [file 484_2020_1921_MOESM1_ESM.docx]

Hot and cold weather based on the Spatial Synoptic Classification and cause-specific mortality in Sweden: A time-stratified case-crossover study

International Journal of Biometeorology

Osvaldo Fonseca-Rodríguez^1,2*^, Scott C. Sheridan^3^, Erling Häggström Lundevaller^2^, Barbara Schumann^1,2^

^1^Department of Epidemiology and Global Health, Umeå University, 901 87 Umeå, Sweden

^2^Centre for Demographic and Ageing Research, Umeå University, 901 87 Umeå, Sweden

^3^Department of Geography, Kent State University, Kent, OH 4242, USA

*** Corresponding author:**Osvaldo Fonseca-Rodríguez
[osvaldo.fonseca@umu.se](mailto:osvaldo.fonseca@umu.se)

**Table S1** Characteristics of each weather type for January and July at each location.

| **Weather**  **type** | **Month** | **Skåne**  **(South West)**  **MMX** | | | | **Stockholm**  **(South East)**  **BMA** | | | | **Jämtland**  **(North West)**  **OSD** | | | | **Västerbotten**  **(North East)**  **UME** | | | |
| --- | --- | --- | --- | --- | --- | --- | --- | --- | --- | --- | --- | --- | --- | --- | --- | --- | --- |
|  |  | % | Ta | Tp | Td | % | Ta | Tp | Td | % | Ta | Tp | Td | % | Ta | Tp | Td |
| **DP** | JAN | 6 | -6 | -5 | -9 | 15 | -9 | -8 | -11 | 12 | -18 | -18 | -21 | 24 | -15 | -15 | -17 |
|  | JUL | 2 | 11 | 17 | 7 | 3 | 12 | 18 | 7 | 5 | 7 | 14 | 4 | 2 | 8 | 14 | 6 |
| **DM** | JAN | 10 | 2 | 2 | -2 | 11 | 1 | 1 | -3 | 13 | -1 | -1 | -6 | 10 | -1 | 0 | -4 |
|  | JUL | 21 | 11 | 21 | 10 | 27 | 12 | 22 | 9 | 15 | 10 | 19 | 7 | 27 | 10 | 19 | 8 |
| **DT** | JAN | 0 | - | - | - | 0 | - | - | - | 0 | - | - | - | 0 | - | - | - |
|  | JUL | 12 | 13 | 25 | 12 | 14 | 15 | 27 | 10 | 9 | 14 | 25 | 9 | 8 | 13 | 24 | 11 |
| **MP** | JAN | 25 | -4 | -3 | -4 | 30 | -4 | -4 | -5 | 31 | -11 | -10 | -12 | 31 | -9 | -9 | -10 |
|  | JUL | 16 | 12 | 15 | 11 | 6 | 11 | 14 | 10 | 11 | 8 | 11 | 6 | 2 | 9 | 11 | 9 |
| **MM** | JAN | 45 | 2 | 3 | 2 | 32 | 1 | 2 | 0 | 33 | -2 | -1 | -3 | 24 | -1 | -1 | -2 |
|  | JUL | 33 | 14 | 19 | 13 | 31 | 14 | 18 | 13 | 43 | 11 | 16 | 10 | 43 | 13 | 17 | 12 |
| **MT** | JAN | 5 | 6 | 7 | 5 | <1 | 6 | 7 | 4 | 0 | - | - | - | 0 | - | - | - |
|  | JUL | 9 | 17 | 24 | 15 | 13 | 17 | 24 | 14 | 9 | 15 | 22 | 11 | 12 | 16 | 22 | 14 |
| **TR** | JAN | 8 | -4 | -1 | -3 | 12 | -4 | -2 | -4 | 11 | -7 | -5 | -8 | 11 | -9 | -5 | -8 |
|  | JUL | 7 | 14 | 19 | 11 | 7 | 14 | 21 | 10 | 8 | 11 | 16 | 8 | 7 | 12 | 18 | 9 |

DP = Dry Polar, DM = Dry Moderate, DT = Dry Tropical, MP = Moist Polar, MM = Moist Moderate, MT = Moist Tropical, TR = Transition, % = Percentage of days in month classified as this weather type, Ta = temperature at 03h (°C), Tp = temperature at 15h (°C), Td = Dew point at 15h (°C). Weather station named in the column of each study location (Malmö—MMX, Bromma airport—BMA, Östersund—OSD and Umeå airport—UME). Detailed information about the characteristics of each weather type regarding the month and location available at <http://sheridan.geog.kent.edu/ssc.html>.


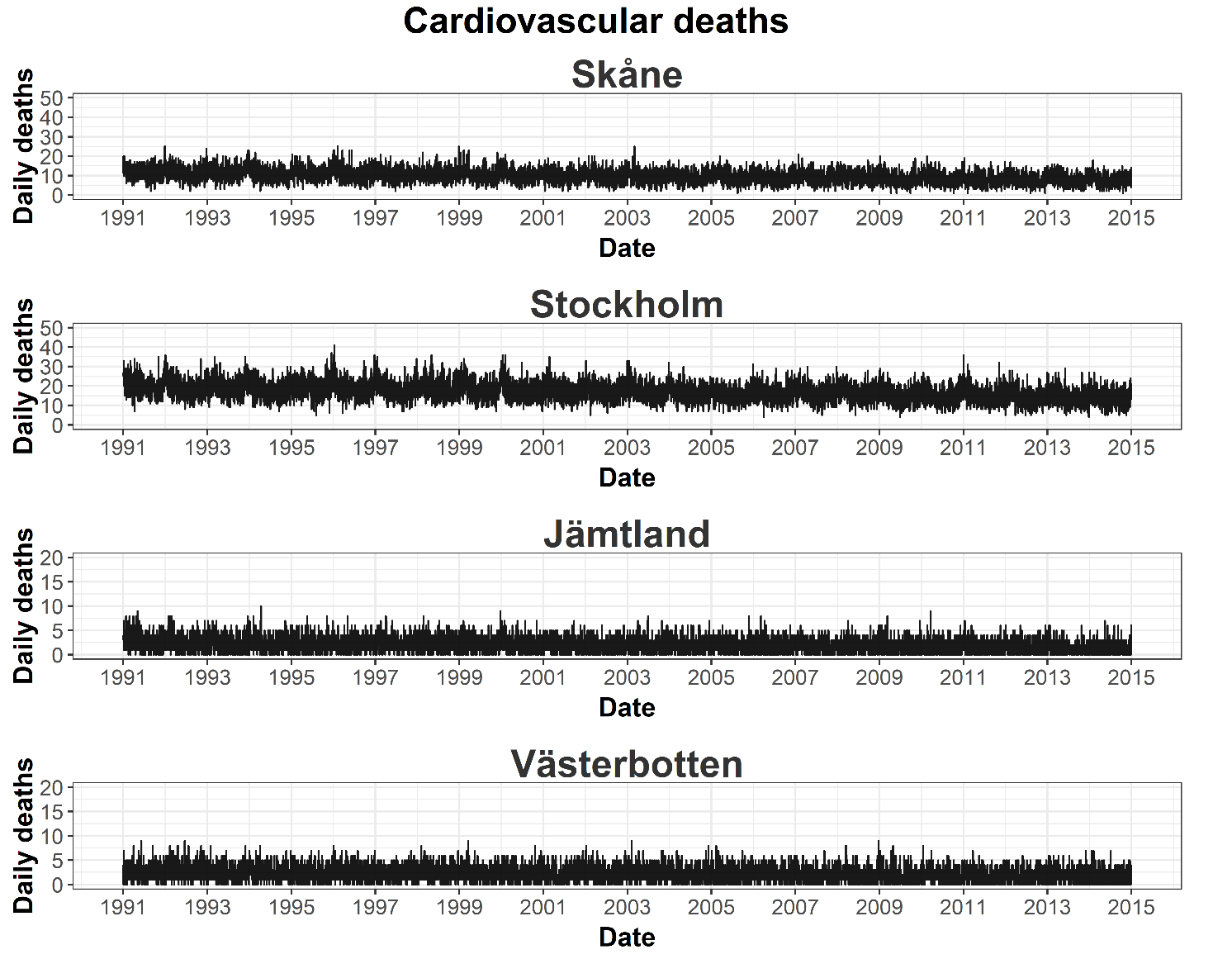


Fig. S1 Time series of daily number of deaths by cardiovascular diseases at each location.


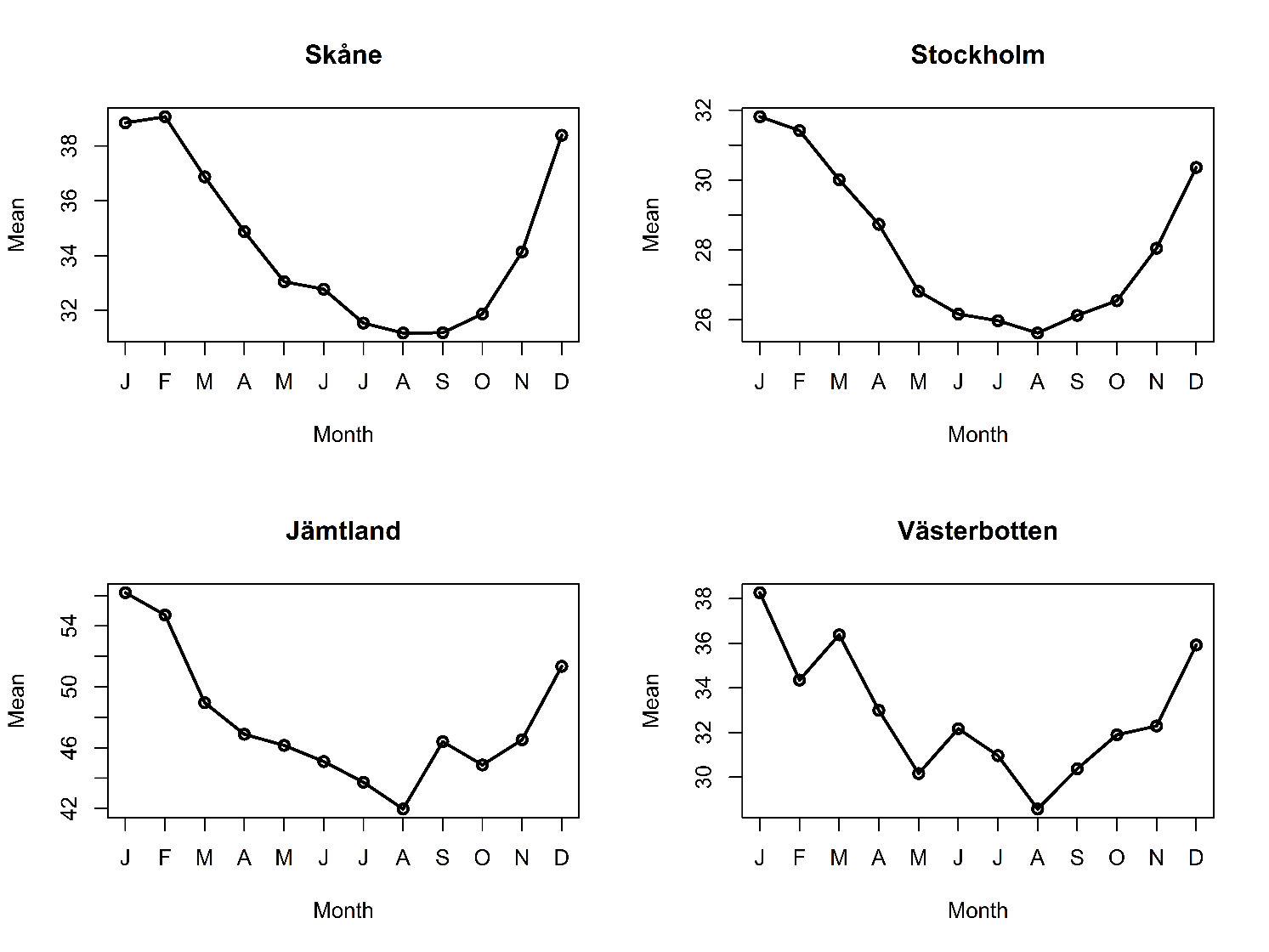


**Fig. S2** Monthly mortality rate (per 100 000 inhabitants) by cardiovascular diseases adjusted for the number of days per month.


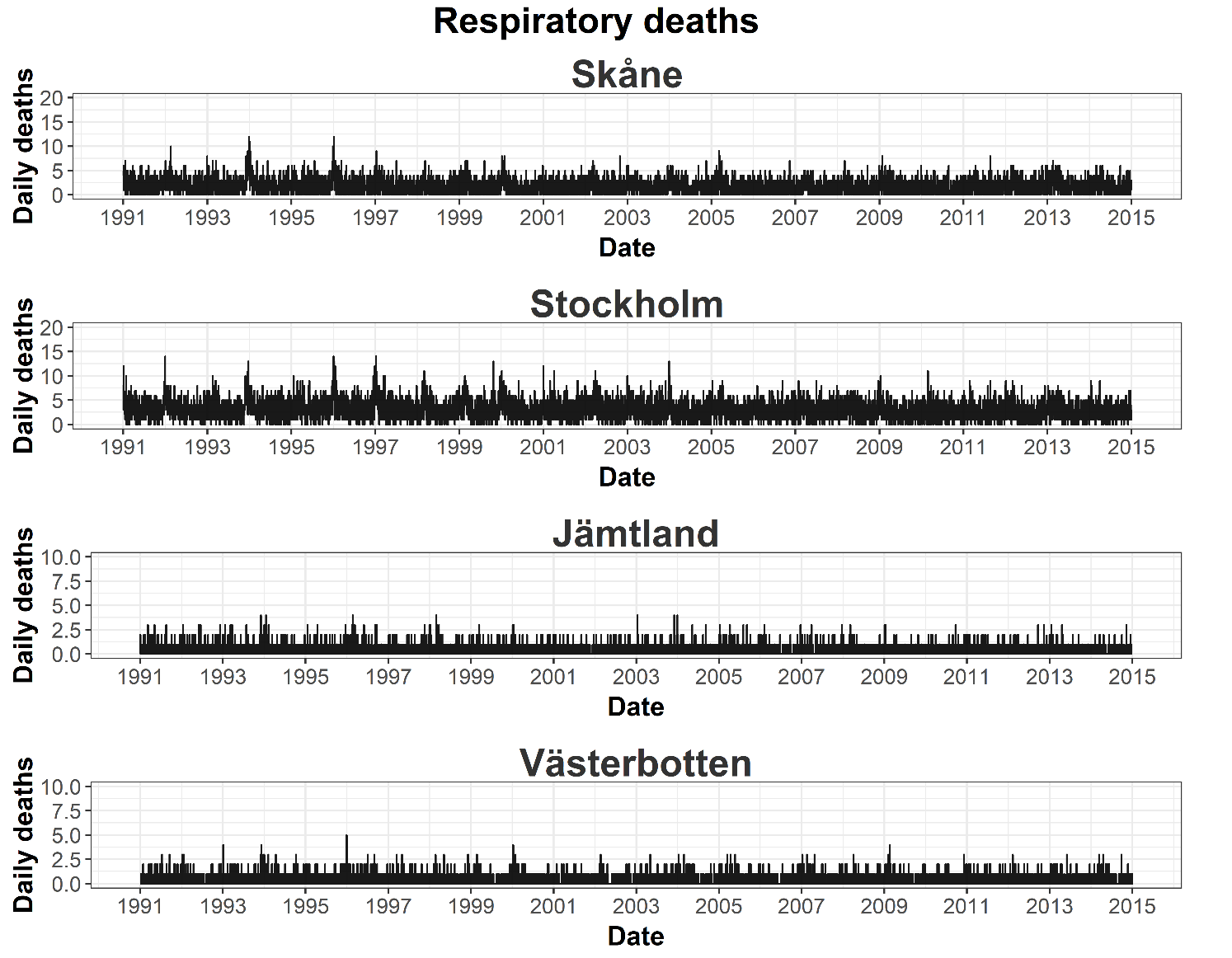


Fig. S3 Time series of daily number of deaths by respiratory diseases in each location: Skåne, Stockholm, Jämtland, Västerbotten.

**
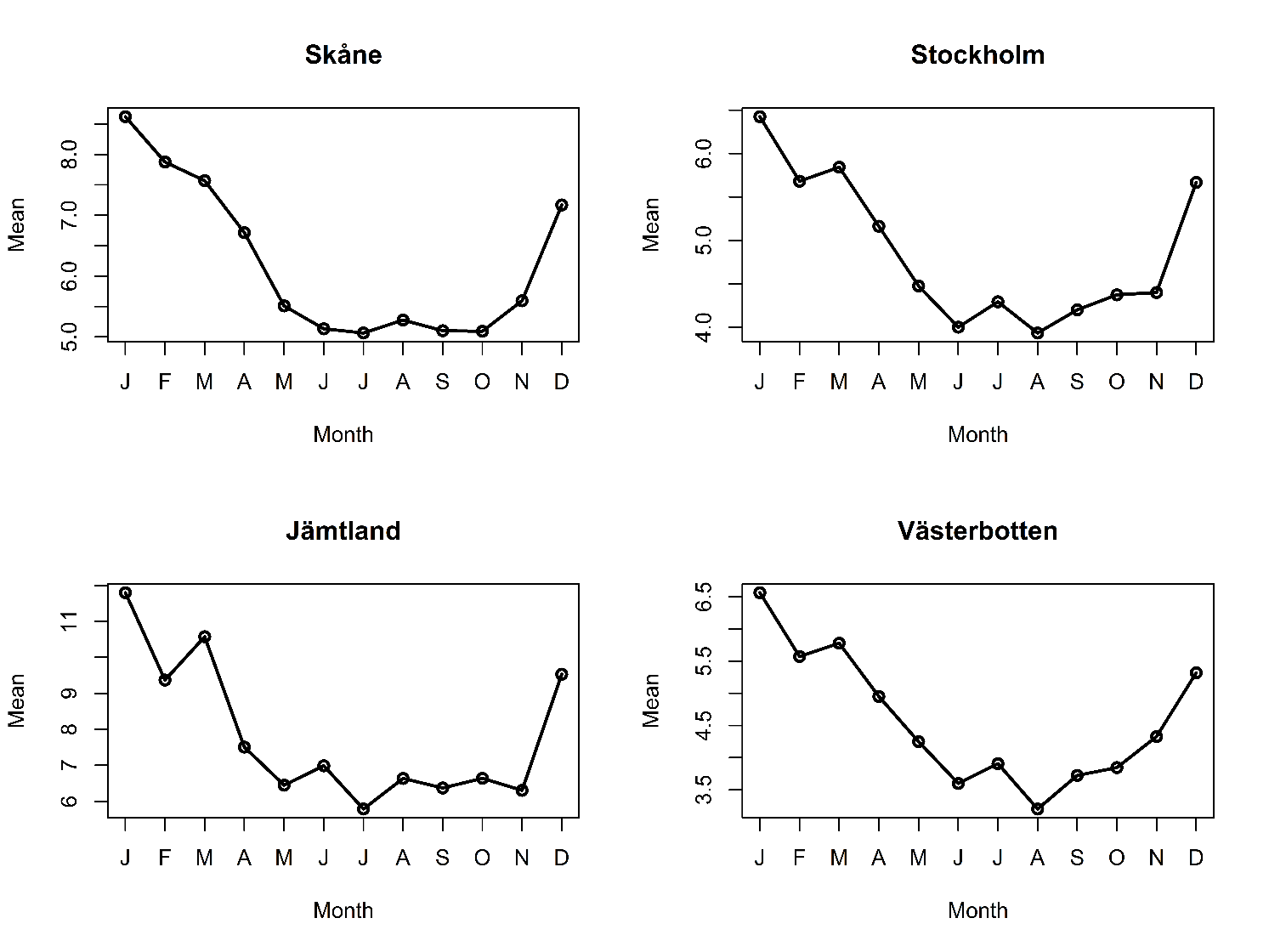
**

**Fig. S4** Monthly mortality rate (per 100 000 inhabitants) by respiratory diseases adjusted for the number of days per month.

**Table S2** Frequency of hot (DT and MT) and cold (DP and MP) days in sequence by location during summer and winter respectively.

| **Season** | **Hot days**  **in sequence** | **Skåne** | **Stockholm** | **Jämtland** | **Västerbotten** |
| --- | --- | --- | --- | --- | --- |
|  |  | **n** | **n** | **n** | **n** |
| **Summer** | 0 | 3057 | 2943 | 3201 | 3183 |
|  | 1 | 227 | 245 | 184 | 201 |
|  | 2 | 118 | 131 | 100 | 98 |
|  | 3 | 81 | 86 | 59 | 61 |
|  | 4 | 48 | 62 | 40 | 35 |
|  | 5 | 32 | 46 | 28 | 23 |
|  | 6 | 23 | 35 | 20 | 17 |
|  | 7 | 20 | 25 | 15 | 15 |
|  | **Cold days**  **in sequence** | **Skåne** | **Stockholm** | **Jämtland** | **Västerbotten** |
|  |  | **n** | **n** | **n** | **n** |
| **Winter** | 0 | 2436 | 1904 | 2190 | 1838 |
|  | 1 | 329 | 423 | 378 | 447 |
|  | 2 | 206 | 270 | 249 | 299 |
|  | 3 | 140 | 198 | 183 | 225 |
|  | 4 | 107 | 151 | 143 | 172 |
|  | 5 | 82 | 115 | 117 | 129 |
|  | 6 | 66 | 98 | 82 | 109 |
|  | 7 | 43 | 68 | 67 | 82 |
|  | 8 | 36 | 57 | 44 | 56 |
|  | 9 | 29 | 45 | 30 | 42 |
|  | 10 | 26 | 38 | 26 | 36 |

**Comparison of conditional Poisson regression model and Time series Poisson regression model**

**Conditional Poisson regression model:**

$$\boldsymbol{Y}_{\boldsymbol{t}}\boldsymbol{\sim}\boldsymbol{quasi}\boldsymbol{-Poisson}\left( \boldsymbol{\mu}_{\boldsymbol{t}} \right)$$

$$\boldsymbol{Log}\left( \boldsymbol{\mu}_{\boldsymbol{t}} \right)\boldsymbol{=\alpha+}\boldsymbol{\beta Crossbasis(Weather types)+}\boldsymbol{\lambda Stratum}_{\boldsymbol{t}}\boldsymbol{+ Offset (log of Population)}$$

As we described in the manuscript: The model outcome was daily deaths ($\boldsymbol{Y}_{\boldsymbol{t}}$) by cardiovascular and respiratory diseases. $\alpha$ is the intercept. Cross-basis matrix of binary variables (Weather types) were created for each of the weather types considered in summer (Dry Tropical—DT and Moist Tropical—MT) and in winter (Dry Polar—DP and Moist Polar—MP). The stratum is an indicator variable composed of year, month, and day of the week (Year:Month:DOW). The count of the total population was interpolated linearly to the daily level by annual population counts for each location. The daily population under risk was used as the offset variable.

**Time series Poisson regression model:**

$$\boldsymbol{Y}_{\boldsymbol{t}}\boldsymbol{\sim}\boldsymbol{quasi}\boldsymbol{-Poisson}\left( \boldsymbol{\mu}_{\boldsymbol{t}} \right)$$

$$\boldsymbol{Log}\left( \boldsymbol{\mu}_{\boldsymbol{t}} \right)\boldsymbol{=\alpha+}\boldsymbol{\beta Crossbasis}\left( \boldsymbol{Weather types} \right)\boldsymbol{+}\boldsymbol{ns}\left( \boldsymbol{year} \right)\boldsymbol{+ ns}\left( \boldsymbol{DOY} \right)\boldsymbol{+\eta DOW+ Offset (log of Population)}$$

In the time series Poisson regression model, a natural spline (*ns*) with 3 degrees of freedom, was used to control for long-term trends (*year*) and seasonal effect within each year (*DOY* – day of the year). Also, a categorical variable *DOW* (day of the week) was included to control for its effect.


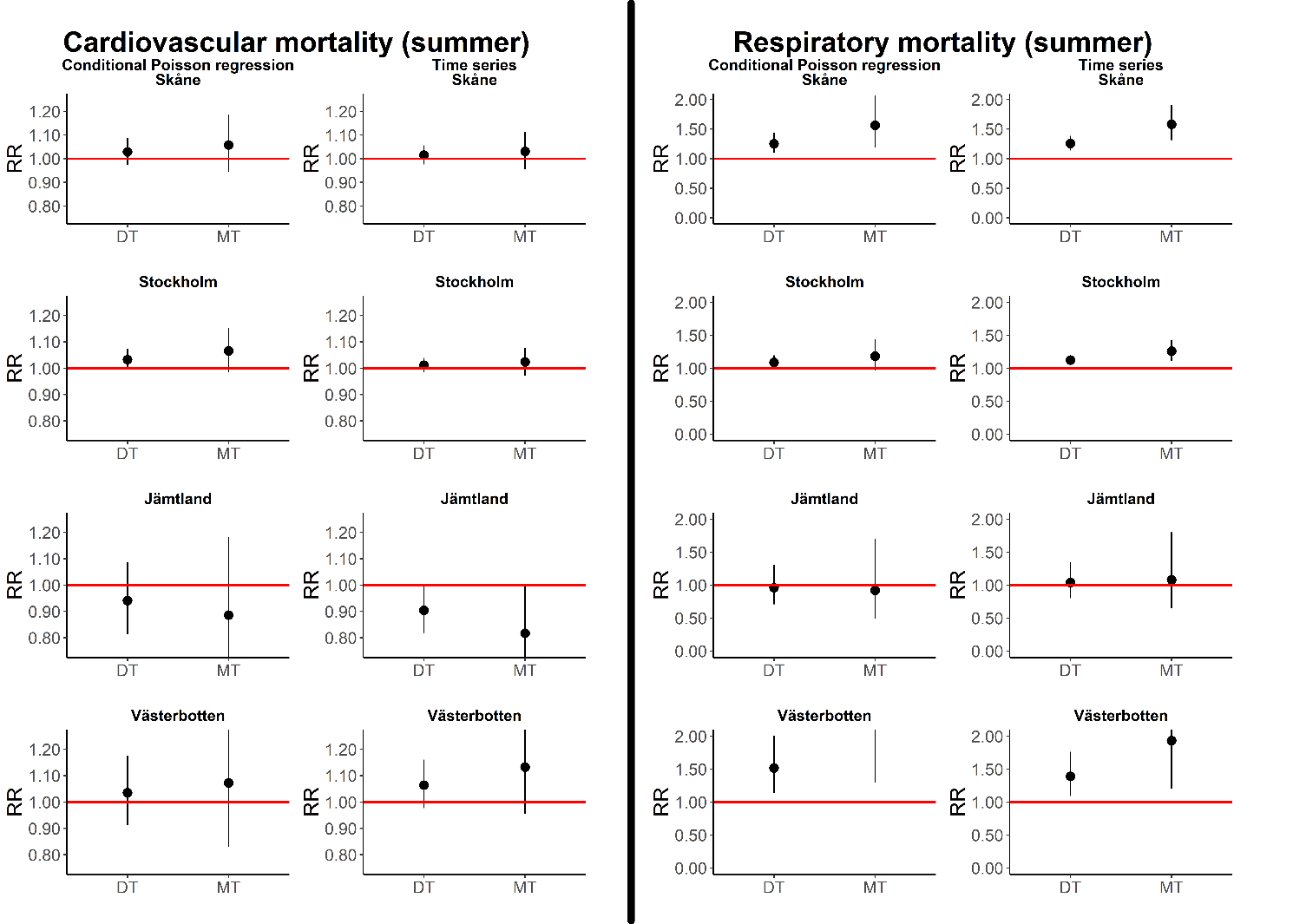


**Fig. S5** Cumulative RR and 95% CI over 14 days of **DT and MT in summer** for cardiovascular (left) and respiratory (right) mortality estimated using conditional Poisson regression and time series analysis.


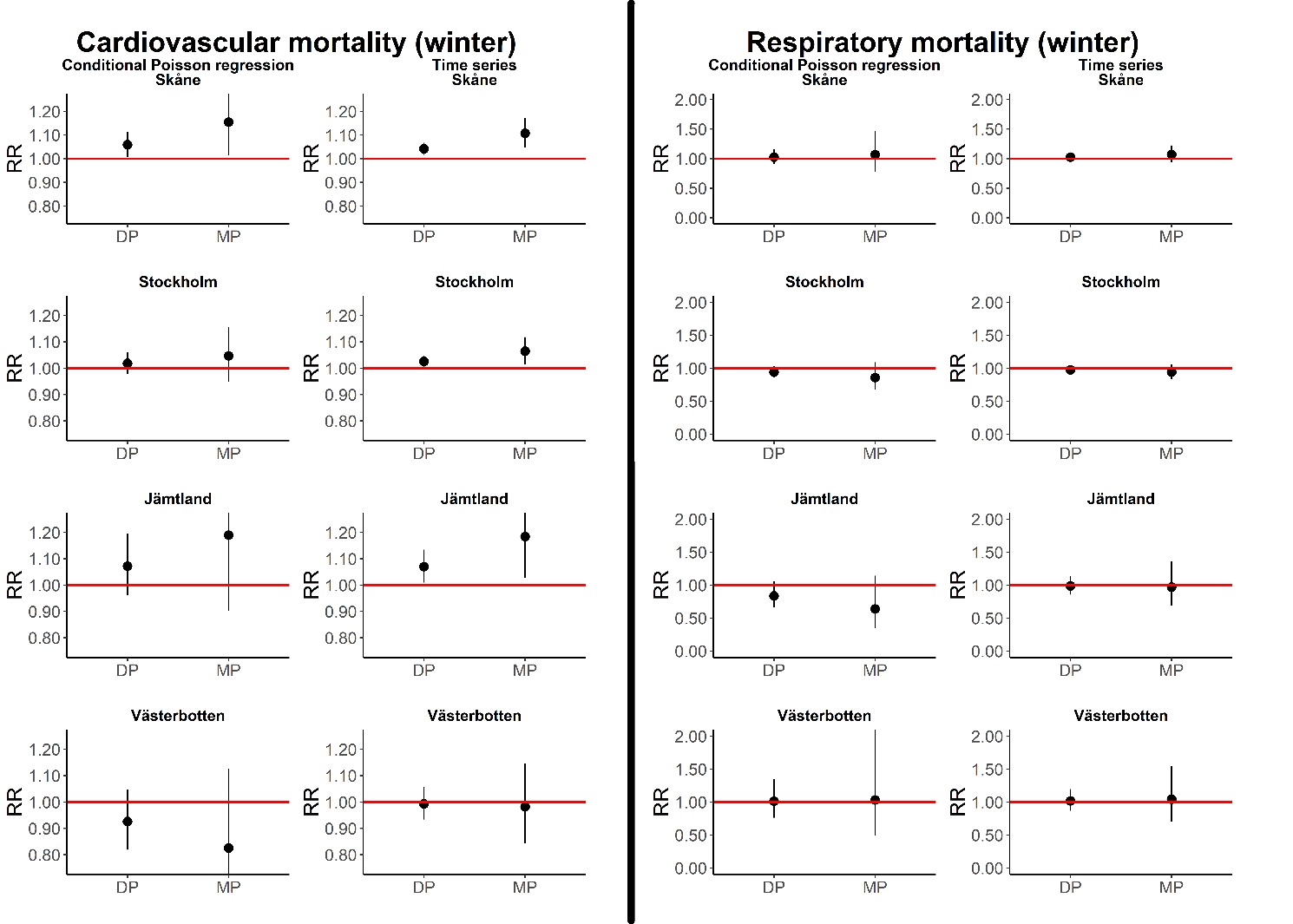


**Fig. S6** Cumulative RR and 95% CI over 28 days of **DP and MP in winter** for cardiovascular (left) and respiratory (right) mortality estimated using conditional Poisson regression and time series analysis.
